# Post quantum blockchain architecture for internet of things over NTRU lattice

**DOI:** 10.1371/journal.pone.0279429

**Published:** 2023-02-01

**Authors:** Bo Yuan, Faguo Wu, Zhiming Zheng

**Affiliations:** 1 School of Computer Science and Engineering, State Key Laboratory of Software Development Environment Beihang University, Beijing, China; 2 Institute of Artificial Intelligence, Beihang University, Beijing, China; 3 Key laboratory of Mathematics, Informatics and Behavioral Semantics (LMIB), Beihang University, Beijing, China; 4 Beijing Advanced Innovation Center for Future Blockchain and Privacy Computing, Beihang University, Beijing, China; 5 Peng Cheng Laboratory, Shenzhen, Guangdong, China; 6 Institute of Medical Artificial Intelligence, Binzhou Medical University, Yantai, China; University of Belgrade Faculty of Organisational Sciences: Univerzitet u Beogradu Fakultet organizacionih nauka, SERBIA

## Abstract

The Internet of Things (IoT) and blockchain, the hottest frontier technologies in recent years, are expected to lead the next technological revolution. Blockchain promises to solve the current challenges encountered by the IoT. However, most of the proposed blockchain-based IoT architectures, which are based on discrete logarithm or large integer factorization problems, are susceptible to quantum attacks. Several quantum-resistant blockchain schemes have recently been proposed. However, the efficiency of their construction or the equipment required is not satisfactory. In this paper, to construct a more efficient postquantum blockchain infrastructure, we propose blockchain architecture for the IoT over the NTRU lattice and provide a cryptographic security proof of the scheme. Attributed to the more efficient underlying lattice structure, our scheme has excellent performance when compared to the existing quantum-resistant blockchain scheme, and we reduce the transaction size from hundreds of megabytes to several kilobytes. To further improve the blockchain’s performance, we present the general framework of segregated witnesses and aggregate signatures over the NTRU lattice. Our scheme promises a blockchain solution for resource-constrained environments.

## Introduction

The Internet of Things (IoT) is a new generation product of the information technology revolution. It is a collection of artificial intelligence, computer, internet, sensor network and other technologies. According to the agreed protocol, the Internet of Things connects any device to a network through information sensing devices to realize intelligent identification, positioning, tracking, monitoring and other functions. By analyzing the information obtained from sensing devices and wireless communications, decision-makers can make a variety of more effective decisions [1, 2]. However, the traditional IoT infrastructure faces many challenges. First, traditional IoT architecture mainly relies on a centralized communication model that connects IoT devices. When the IoT network becomes more extensive, this model is unlikely to expand. Second, a single bottleneck may cause an interruption of the whole IoT network [3]. In the end, due to the long data transmission time, IoT communication latency will increase considerably due to network structure, thus hindering the large-scale deployment of the IoT in actual scenarios [4]. Sharma et al. presented the security solutions inherent to securing the IoT environment and discussed steps that should be taken to make a safe IoT environment [5].

Blockchain, initially used as technical support for the digital currency, Bitcoin, has been another popular topic in recent years and can offer a scalable and decentralized environment for IoT devices, platforms, and applications, combining IoT and blockchain toward new levels of trust. The IoT market will reach 5802.7 million dollars by 2026 from 32 million dollars in 2018 [6]. Thereafter, researchers proposed some constructive blockchain-based architectures for the IoT. Md. Abdur Rahman et al. use blockchain and IoT to build a sharing economy system to support secure smart city services (MEC) [1]. Arsyad et al. proposed blockchain based Encapsulating Block Mesh (EBM) to eliminate data tampering and distortion, allowing data to be monitored safely and transparently [7]. Raj et al. proposed a blockchain-based access control for a healthcare monitoring system which can reduce deployment and execution latency and average response latency in the real-time smart healthcare system [8]. Gupta et al. proposed a very efficient attribute-based searchable encryption scheme for healthcare cloud-based cyber-physical system with the assistance of consortium blockchain [9]. Lu et al. constructed a novel blockchain-based cloud storage protocol for sensors in Industrial Internet of Things which can realize secure data sharing with a less computational overhead [10]. In 2019, Sheng Ding et al. proposed a novel attribute-based access control scheme for the Internet of Things that simplifies access management [11]. Pradip Kumar Sharma et al. proposed a distributed blockchain cloud architecture model that can effectively offload data to the cloud [12]. Different blockchain-based architectures validate that the combination of blockchain and IoT has great potential.

### Related works

The abovementioned blockchain-based IoT architectures are based on traditional number theory problems, which are insecure against quantum analysis [13]. Postquantum cryptography is a new generation of cryptographic algorithms that can resist quantum computers and is expected to gradually replace current public key cryptographic algorithms, such as RSA, Diffie-Hellman, and elliptic curves in the next 5-10 years. Researchers have done a number of creative things to make blockchain resistant to quantum computer attacks. Kiktenko et al. proposed a quantum-secured blockchain based on quantum key distribution and methodology [14]. Gerardo Iovane’s MuReQua Chain is also based on quantum networks [15]; however, quantum key distribution networks are not compatible with traditional networks and are too costly. Some researchers replace the underlying blockchain signature algorithm with a lattice-based signature algorithm, which provides a foundational framework for quantum-resistant blockchain design [16–19]. While these solutions have produced very valuable results, they may not be applicable to resource-constrained environments, such as the IoT, as their lattice-based signatures take up too much space and the performance of the blockchain drops dramatically. Compared with signatures based on traditional number theory problems, lattice-based signature schemes have one serious disadvantage: the efficiency of existing lattice signature schemes (especially communication efficiency) is relatively low.

Is it just a matter of replacing the traditional cryptographic scheme in the blockchain with a quantum-resistant cryptographic scheme? If we only analyze the postquantum signature scheme from the cryptographic level, we generally consider the security level, signature size and speed, public key size and speed, and private key size and speed. If we look at the postquantum cryptographic scheme from the application level, in addition to the points mentioned above, we must also consider the availability of specific implementation codes, stateful/stateless, and the maximum limit on the number of signatures. Therefore, for our IoT application scenarios, under the premise of satisfying security and practicability, the postquantum cryptographic scheme investigation factors are divided into performance requirements and space requirements. Unfortunately, the signatures of postquantum cryptographic schemes are often tens or even hundreds of times longer than those of traditional cryptographic schemes [20].

The rapid development of blockchain technology leads to the demand for high-quality applications based on blockchain. This poses a key challenge to the design of a high-performance blockchain protocol because the performance of a blockchain network ultimately depends on the chosen consensus mechanism. For a long time, one of the main challenges of blockchain technology was how to improve throughput, i.e., how to improve the transaction speed. The only way to achieve this improvement is to first understand the cause of the bottleneck.

Most IoT devices are embedded terminals or sensors with low computing and storage capabilities [21]. Especially for some devices that use portable energy, energy consumption affects the life of the entire network. The traditional blockchain consensus algorithm based on the workload proof mechanism is not suitable for the Internet of Things scenario. When considering the combination of the blockchain and the internet, the existing blockchain systems have limited throughput. For example, the Bitcoin system can only process 7 transactions per second, and Ethereum can only process 15 transactions per second on average. In the Internet of Things scenario, improving system throughput is the premise of applying blockchain technology. Sun et al. [22] emphasizef that system throughput is a key indicator that affects blockchain performance and affects the optimal full-function node deployment strategy of the system.

### Motivation

Blockchain security mainly comes from consensus mechanisms and asymmetric cryptosystems (digital signatures). Quantum computers cannot produce a substantial threat to the consensus mechanism and to Bitcoin, the POW consensus mechanism; for example, the POW is actually looking for the preimage of the hash function, SHA256, with a specific output length, and quantum computers can indeed accelerate the speed of computing the hash. However, compared to the brute force key search, Grover’s algorithm can only achieve a square root acceleration [23]. This means that to guarantee the security of these types of algorithms in the blockchain, it is only necessary to make the algorithm output correspondingly long.

The greatest threat of quantum computers to blockchain is the asymmetric cryptosystem (digital signature). The current digital signature of a blockchain system is basically based on the elliptic curve digital signature algorithm (ECDSA), and the mathematics behind it is the elliptic curve discrete logarithm problem (ECDLP), which is difficult to solve using classical computers. Under the classical computer model, it is exponentially difficult to solve ECDLP, and under the quantum computing model, it is polynomial to solve this problem, thus making the whole signature system no longer secure.

Cryptocurrency addresses are created by hashing or masking the public key. When a user makes a transaction, the public key is exposed on the blockchain. Satoshi Nakamoto has cleverly used double hashing in Bitcoin. Interestingly, double hashing not only hides the real public keys of the nodes but also makes Bitcoin resistant to quantum attacks as long as each node changes its address after every transaction. However, very few users change their address after each transaction.

The biggest impact of quantum computers on the blockchain is that hackers can easily exploit the flaws in the current blockchain system authentication and use the victim’s exposed account in the network to obtain the user’s private key to generate new transactions, which has a devastating impact on the blockchain system [13].

Blockchain is expected to play an important role in the future, with asymmetric cryptosystems as its trusted foundation. History teaches us that technology changes faster than we expect and often in a nonlinear fashion. Postquantum construction must be taken into consideration.

### Contributions

Inspired by the researches and analyses [16–19, 24], we change the underlying cryptographic structure of the quantum-resistant blockchain, the main contributions in this paper are as follows:

(1) We present the advantages of combining blockchain and IoT and analyze the flaws of the current blockchain that cannot resist quantum attacks. In addition, we give an analysis of the bottlenecks of the current postquantum blockchain research.
(2) We construct the post quantum blockchain architecture for Internet of Things over NTRU Lattice which can ensure blockchain system is compatible with existing classical channels. Furthermore, we generate the wallet seed key over NTRU lattice and use seed key to generate the subpublic keys and subprivate keys which guarantees the randomness of the key and the lightness of the wallet.
(3) We present the correctness of our scheme and prove our scheme is existential unforgeable against the adaptive chosen message and address attacks over *γ*-shortest vector problem on the NTRU lattice which guarantee the security of our scheme under the quantum computing model.
(4) Compared with the existing quantum-resistant blockchain scheme, our scheme is considerably improve quantum-resistant blockchain performance, we reduce the transaction size from hundreds of megabytes to several kilobytes.
(5) We analyze the impact of transaction size on blockchain performance and provide two effective options for improving the performance of the post quantum blockchain. The work in this paper helps enrich the lattice-based postquantum blockchain.

### Organization

The paper is organized as follows: In Section 2, we review the advantages of combining blockchain and IoT and analyze the flaws of the current blockchain that cannot resist quantum attacks and the bottlenecks of the current postquantum blockchain research. In Section 3, we introduce some of the a priori knowledge needed to construct a postquantum blockchain over the NTRU lattice. In Section 4, we present the seed key generation algorithm and the corresponding postquantum blockchain specific construction method. In Section 5, we compare our scheme with the latest lattice-based blockchain. In Section 6, to further improve the performance of the quantum-resistant blockchain, we present two improvement options.

## Blockchain, IOT and quantum computer

### IoT and blockchain

**Fig 1:**
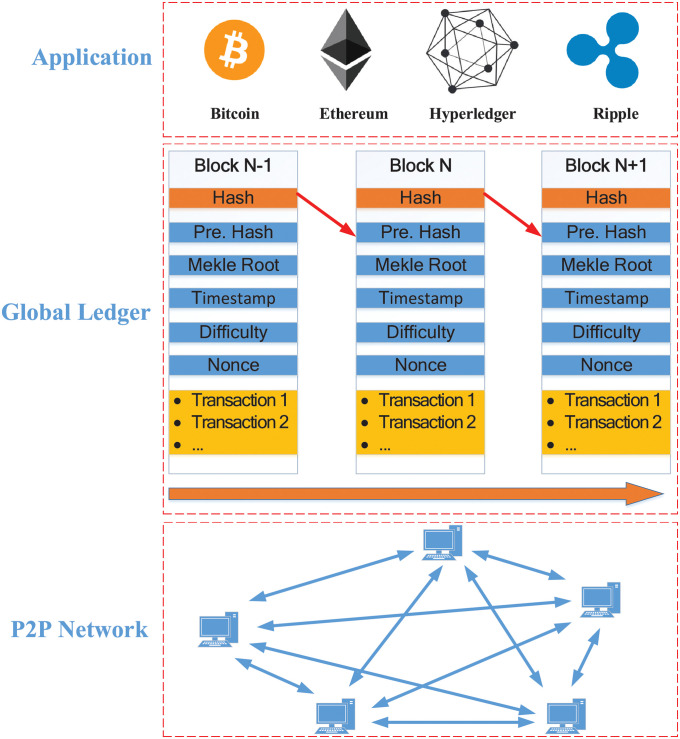
Blockchain structure of Bitcoin.

Blockchain technology is an advanced distributed database mechanism consisting of a combination of cryptography, consensus mechanisms and other technologies that allow users to share information transparently across the network, and messages are not allowed to be tampered with once they are on the chain. It provides full lifecycle protection for data. The underlying blockchain technology structure of Bitcoin is shown in Fig 1.

**Fig 2:**
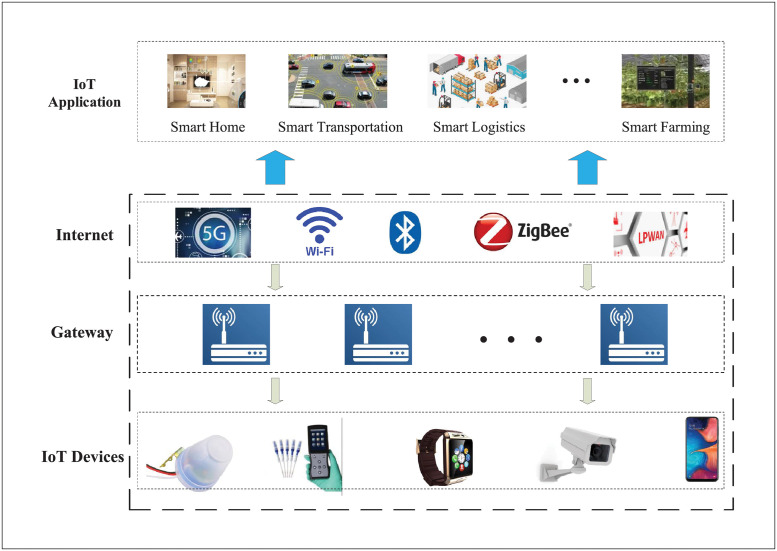
Traditional architecture of IoT.

The traditional internet-based IoT architecture faces data privacy and security issues. Traditional architecture of the Internet of Things is shown in Fig 2. The decentralized autonomy, tamper-proof and security features of blockchain technology can bring changes to many conveniences in the field of IoT and provide new ideas for the challenges faced by IoT.

- Defense Against DDOS: With the explosion of smart cities and the Internet of Things, there has been an exponential surge in IoT devices in recent years. Hundreds of millions of IoT terminal devices are connected to each other through edge nodes, automating daily tasks and delivering data to central servers. However, the massive growth in the number of IoT devices has also made IoT networks more vulnerable to DDoS attacks. Malware, such as Mirai [25] can infect and spread unsafe IoT devices. In a distributed environment, it is very easy to infect the entire network, forming a network of zombie devices to attack servers. The distributed P2P network architecture based on blockchain can monitor and audit the spread of network data, and any transaction needs to be authenticated. Therefore, it can greatly control the spread of traffic carrying dangerous viruses in the Internet of Things. Javaid et al. [26] supplanted the conventional centralized IoT architecture with a distributed IoT architecture based on Ethereum and smart contracts. All IoT devices use smart contracts to access the network and solve DDoS by using static resource allocation of device attacks. Chen et al. [27] studied the DDoS threat in an IoT network and offered a solution based on blockchain. Their solution first removes the network traffic data of edge nodes, examines the data characteristics, and checks the abnormal behavior of terminal devices. Finally, according to the characteristics of the attack node traffic data, the corresponding access control strategy is formulated, and smart contracts are deployed to achieve DDoS attack defense.
- Privacy Security: The IoT is closely related to people’s daily lives, and the massive data information carried by various sensors and communication equipment has also caused people to worry about privacy and security. Through the use of blockchain technology, people can realize the safe storage and controllable management of data. Aiming at the problem that access control is difficult to deploy in IoT networks, Ouaddah et al. [28] used blockchain to realize access control of restricted devices in IoT. They developed a decentralized privacy authorization framework that is anonymous. Yang et al. [29] developed an interactive energy management system based on blockchain to address the problems of privacy leakage and a single point of failure in traditional energy transaction management. Kumar et al. [30] designed an enhanced consensus mechanism based on Ethereum to authenticate IoT data records and prevent data poisoning from threatening the entire IoT data security. Liu et al. [31] combined searchable encryption, attribute encryption, blockchain and other technologies and proposed a new management mechanism to manage IoT data, enabling the system to have controllable data management authority and ciphertext data retrieval capabilities.
- Deploy Smart Contracts: Smart contracts are a method of using blockchain to implement agreements between parties. By using encryption algorithms and other blockchain security mechanisms, once the smart contract is deployed, it automatically executes the predetermined content of the agreement, and all states during the execution of the smart contract are observable. Therefore, smart contracts provide better security than traditional contracts and reduce other transaction costs associated with contracts, and the smart contract blockchain can flexibly implement IoT application functions. Pan et al. [32] constructed an edge IoT framework, EdgeChain. EdgeChain integrates a permissioned chain and smart contracts, regulates resource acquisition and use behavior through an internal currency system, and regulates resource management systems by formulating a credit system. For the transparency of execution, all states are recorded in the blockchain, thus achieving secure recording and auditing of data. Huh et al. [33] built an IoT device management system based on the Ethereum blockchain, stored RSA public and private key information on the chain and on each IoT device, respectively, and used a smart contract written in the Turing-complete language to manage IoT device configurations and build key management systems.
- Data Integrity: A major challenge in IoT data management is ensuring data integrity. Usually, the IoT needs to collect data from edge devices, such as smart home devices, industrial sensors, and smart cameras and upload it to the cluster computing resource center for data modeling and analysis. Therefore, it is very important to ensure that the data collected by IoT devices are complete and reliable. Due to the decentralized nature of the IoT structure, data may need to be transferred between multiple nodes, ensuring that the data are not tampered with or poisoned during the transfer process, and maintaining integrity has become a challenge for IoT applications. In the absence of third-party audits, Liu et al. [34] proposed a service framework for verifying data integrity based on blockchain. In the Internet of Things environment, this service can verify the integrity of the data in the transmission process for data owners and data users. Zhao et al. [35] constructed a data integrity checking scheme based on blockchain, bilinear pairing and the elliptic curve ElGamal encryption algorithm. Using aggregated signature technology based on bilinear pairing, batch signature verification was realized, and the data verification efficiency in the Internet of Things was improved.
- Supply Chain Management: Supply chain management plays an important role in improving the overall efficiency of the industry. By strengthening supply chain management, the entire circulation process of commodities can be fully optimized, and the circulation path of commodities can be shortened. Efficient supply chain management has become an important source of competitive advantage. However, it is difficult to verify the origin of raw materials and keep them open and transparent as products and commodities move through the value chain network.

Applying blockchain can help enterprises overcome the problems of data collection and data integrity, better realize the traceability of data, and reduce the problem of information asymmetry among all parties in the industrial chain [36]. The application of IoT devices can help participants in the industry chain monitor the entire process of product production, transportation, registration, and sales in the industry chain network.

## Preliminaries

### Notations

In this paper, the following notations are used:

- *N* is a security parameter with a power of 2.
- ∥*x*∥ indicates the Euclidean norm of *x*.
- *x* ∥ *y* indicates the concatenation of two strings *x* and *y*
-
$R_{q}=Z_{q}/\left(X^{N}+1\right)$ indicates the ring of polynomials modulo *X*^*N*^ + 1 with coefficients in $Z_{q}$.

### NTRU lattice

*N* is a power of 2, *q* > 0, and compute polynomials *h* as follows:

$$\begin{array}{c} h=f^{-1}g\text{mod}\left(X^{N}+1\right) \end{array}$$ (1)

The NTRU lattice associated with *h* and *q* is defined as follows:

$$\begin{array}{c} \underset{h,q}{⋀}=\left\{\left(u,v\right):u+v*h\;\text{mod}\;q=0\right\} \end{array}$$ (2)

${⋀}_{h,q}\in Z^{2N}$ and generated by the rows of

$$\begin{array}{c} A_{h,q}=(\begin{array}{cc} A_{N}\left(h\right) & I_{N}\\ qI_{N} & O_{N} \end{array}) \end{array}$$ (3)

$A_{N}\left(h\right)$ is an anticirculant matrix whose *i*^*th*^ row consists of the coefficients of the polynomial *hx*^*i*^ mod (*X*^*N*^ + 1).

**Definition 1** *The ′q − ary′ lattice is defined as follows*:

$$\begin{array}{c} \begin{array}{c} \Lambda_{q}\left(A\right)=\left\{S\in Z^{m}:x=A^{T}S=u\left(\text{mod}q\right)\right\}\\ \Lambda_{q}^{⊥}\left(A\right)=\left\{S\in Z^{m}:x=A^{T}S=0\left(\text{mod}q\right)\right\} \end{array} \end{array}$$

### Discrete gaussian distribution

The Gaussian series, the most important component of the lattice cipher, is widely used in lattice signature schemes and is defined as follows:

**Definition 2** $s\in R^{m}$ *is the standard deviation, vector* $c\in Z^{m}$ *is the center, and the Gaussian function is defined as follows*:

$$\begin{array}{c} \begin{array}{c} g_{s,c}\left(x\right)=e^{\frac{-\pi^{2}∥x-c{∥}^{2}}{2s^{2}}} \end{array} \end{array}$$ (4)

*The discrete Gaussian distribution over* Λ *with center* **c** *and parameter* **s** *is defined as follows*:

$$\begin{array}{c} \begin{array}{c} G_{\Lambda ,s,c}\left(x\right)=\frac{g_{s,c}\left(x\right)}{\sum_{x\in \Lambda }g_{s,c}\left(x\right)} \end{array} \end{array}$$ (5)

**Definition 3** *The continuous normal distribution is defined as follows*:

$$\begin{array}{c} \begin{array}{c} \rho_{\sigma ,c}^{m}\left(x\right)={\left(\frac{1}{\sqrt{2\pi \sigma^{2}}}\right)}^{m}e^{\frac{-∥x-c{∥}^{2}}{2\sigma^{2}}} \end{array} \end{array}$$ (6)

$\sigma \in R^{m}$ *is the standard deviation, and* $c\in Z^{m}$ *is the center*.

*The discrete normal distribution is defined as follows*:

$$\begin{array}{c} \begin{array}{c} D_{\Lambda ,\sigma ,c}^{m}\left(x\right)=\frac{\rho_{\sigma ,c}^{m}\left(x\right)}{\sum_{x\in Z^{m}}\rho_{\sigma ,c}^{m}\left(x\right)} \end{array} \end{array}$$ (7)

*When* **c** = 0, $D_{\sigma ,c}^{m}\left(x\right)$ *and* $\rho_{\sigma ,c}^{m}\left(x\right)$ *are simply noted as* $D_{\sigma }^{m},\rho_{\sigma }^{m}$.

When the discrete normal distribution has a standard deviation *σ* in dimension *m*, we can obtain some important properties of the discrete Gaussian distribution [37].

**Lemma 1** ∀*σ* > 0 *and* $m\in Z^{+}$

*(1)* $Pr\left[x\in D_{\sigma }^{1}:|x|>12\sigma \right]<2^{-100}$ ;

*(2)* $Pr\left[x\in D_{\sigma }^{m}:∥x∥>2\sigma \sqrt{m}\right]<2^{-m}$ ;

**Lemma 2** *If* $\sigma =\omega (∥v∥\sqrt{\text{log}m})$ , *then ω*(.) *is the nonasymptotic tight lower bound, and we obtain the following*:

$$\begin{array}{c} Pr\left[x\in D_{\sigma }^{m}:D_{\sigma }^{m}\left(x\right)/D_{\sigma ,v}^{m}=o\left(1\right)\right]=1-2^{\omega \text{log}m} \end{array}$$ (8)

*More specifically, when σ = α* ∥ *v*∥, *the probability can be derived as follows*:

$$\begin{array}{c} Pr\left[x\in D_{\sigma }^{m}:D_{\sigma }^{m}\left(x\right)/D_{\sigma ,v}^{m}<e^{12/\alpha +1/(2\alpha^{2})}\right]>1-2^{-100} \end{array}$$ (9)

The above Lemma holds for any positive real *α* and $v\in Z^{m}$.

### GaussianSampler

We can obtain vectors that follow a discrete Gaussian distribution by the following algorithm:

**Algorithm 1** SampleGau

**Input:** Lattice ⋀ basis *B*, standard deviation *σ*, center $c\in Z^{N}$

**Output:** Vector *v* sampled in *D*_Λ,*σ*,*c*_

1: *v*_*n*_ ← 0

2: *c*_*n*_ ← *c*

3: **for** *i* = *n*, *n* − 1, …, 1 **do**

4:  $c_{i}^{{}^{\prime }}\leftarrow \langle c_{i},\tilde{b_{i}}\rangle /{∥\tilde{b_{i}}∥}^{2}$

5:  $\sigma_{i}^{{}^{\prime }}\leftarrow ∥\tilde{b_{i}}∥$

6:  $z_{i}\leftarrow SampleZ\left(c_{i}^{{}^{\prime }},\sigma_{i}^{{}^{\prime }}\right)$

7:  *c*_*i*−1_ ← *c*_*i*_ − *z*_*i*_*b*_*i*_

8:  *v*_*i*−1_ ← *v*_*i*_ − *z*_*i*_*b*_*i*_

9: **end for**

10: **return** *v*_0_

**SampleZ** is a subalgorithm that samples a 1-dimensional Gaussian $D_{Z,\sigma^{{}^{\prime }},c^{{}^{\prime }}}$, and we can obtain it by rejection sampling and look-up tables.

### Rejection sampling technique

The prerequisite for the security of the signature algorithm is to eliminate the relationship between the key and the output signature, which we can achieve in the lattice signature with the following rejection sampling algorithm.

**Algorithm 2** Rejection Sampling Technique

**Input:** *u* is the message, matrix *A* is randomly sampled from $Z_{q}^{m\times n}$, and **S** is sampled from {−*d*, …, 0, …, *d*}^*m*×*k*^, *H*: {0, 1}* → {*v*: *v* ∈ {−1, 0, 1}^*k*^, ∥*v* ∥ <*κ*}, where *d* ≪ *q*^*n*/*m*^, k∈Z and ≪*m*, *κ* is constant and 2κ·(kκ)≥2100. Then, there exists a constant *M* = *O*(1).

**Output:** Vector **z** and **c**

1: Obtain **y** randomly from $D_{\sigma }^{m}$

2: **c** = *H*(*A***y**, *u*)

3: **z** = **Sc** + **y**

4: **return** (**z**, **c**) with probability $min(\frac{D_{\sigma }^{m}\left(z\right)}{MD_{Sc,\sigma }\left(z\right)},1)$

### Hardness assumption

The hardness assumption is a theoretical guarantee for the security of cryptographic schemes, and the *γ*-shortest vector problem on the NTRU lattice guarantees the security of our scheme under the quantum computing model. The cryptographic security hardness assumption involved in our scheme is as follows:

**Definition 4** *(SIS problem) Given a matrix* $A\in Z_{q}^{m\times n}$ , *a prime q and a real β, the SIS problem finds a nonzero vector* $e\in Z^{m}$ *that satisfies* **Ae** = 0 *mod q and* ∥**e** ∥ <*β*.

**Definition 5** *Given f*, *g*, *h as involved in Algorithm 3, a positive q and a real β, the SIS on the NTRU lattice is to find a nonzero vector* (*z*_1_, *z*_2_) *that satisfies* **A**_*h*,*q*_(*z*_1_, *z*_2_) = 0 *mod q and* ∥(*z*_1_, *z*_2_)∥ < *β*.

Assume that (*s*_1_, *s*_2_) is any of the vectors in the *A*_*h*,*q*_, the *γ* − *SVP* problem on the *A*_*h*,*q*_ is to find the vector (*z*_1_, *z*_2_) satisfy∥(*z*_1_, *z*_2_)∥ ≤ *γ* ∥ (*s*_1_, *s*_2_)∥, that is, ∥(*z*_1_, *z*_2_)∥ ≤ *γϑ*. *ϑ* is the shortest vector in *A*_*h*,*q*_. We let *γ* = *β*/*ϑ*. When the approximate factor *γ* < 1 + 1/*n*^*ε*^, the *γ*-shortest vector problem is NP-hard [38].

## Our construction

According to our previous analysis, to build a blockchain that can resist quantum computers, it is necessary to ensure that the cryptographic algorithms used in the underlying blockchain are secure under the quantum computing model. Therefore, we replace the current signature scheme in the blockchain with a lattice-based signature. The parameters involved in our scheme need to satisfy the following range of values

(1)*M*, m>5nlogq, *q* ≥ 3.
(2)$\tilde{L}=O\left(\sqrt{n\text{log}q}\right)$, $s=\tilde{L}•\omega \left(\sqrt{\text{log}n}\right)$. *σ* = 12λ*sm*.
(3)*H*_1_ = {0, 1}* → {**v**: **v** ∈ {−1, 0, 1}^*k*^, ∥**v** ∥ ≤λ}, which is modeled as a random oracle.

### Wallet seed key generation

The node generates the seed key of the wallet according to the security parameters, the seed key is stored in the deterministic wallet, all the secret keys of the node are generated by the seed secret key, and the node only needs to make a simple storage backup at the beginning creation stage. When a node wants to sign a transaction with its private key, the node uses the seed key to generate all the private keys. The idea of the seed secret key is essentially the same as the idea of KGC; that is, we let the node itself become weakly central. The seed key generation algorithm is shown in Algorithm 3.

**Algorithm 3** Seed Key Generation

**Input:** Security parameter *N*, prime *q*, *σ*

**Output:** Public key *mpk* and secret key *msk* of KGC.

1: **Start** Sample *f*, *g* ∈ *D*_*Z*^*N*^, *σ*_.

2: **if** $∥f∥>\sigma \sqrt{N}$ or $∥g∥>\sigma \sqrt{N}$ or *f*mod*q* $\notin R_{q}^{*}$ or *g*mod*q* $\notin R_{q}^{*}$ **then**

3:  **Restart**

4: **end if**

5: **if** max($∥\left(g,-f\right)∥,∥\left(\frac{q\bar{f}}{f\bar{f}+g\bar{g}},\frac{q\bar{g}}{f\bar{f}+g\bar{g}}\right)∥)>1.17\sqrt{q}$ **then**

6:  **Restart**

7: **end if**

8: *R*_*f*_ = *resultant*(*f*, *X*^*N*^ + 1) and *R*_*g*_ = *resultant*(*g*, *X*^*N*^+ 1) respectively. The resultant of *f* can be straightforwardly calculated as $\prod_{i=1}^{N-1}f\left(X^{i}\right)$ (mod *Φ*(*N*)) where *Φ*(*N*) is cyclotomic polynomial *Φ*(*N*) = 1 + *X* + *X*^2^ + … + *X*^*N*−1^. The details of the *resultant* operation can refer to [39]

9: Compute *ρ*_*f*_, *ρ*_*g*_ satisfy *ρ*_*f*_*f* + *k*_*f*_(*X*^*N*^ + 1) = *R*_*f*_, *ρ*_*g*_*g* + *k*_*g*_(*X*^*N*^ + 1) = *R*_*g*_ by the Extended Euclidean Algorithm where *k*_*f*_ and *k*_*g*_ are integer.

10: **if** *GCD*(*R*_*f*_, *R*_*g*_) ≠ 1 or *GCD*(*R*_*f*_, *q*) ≠ 1 **then**

11:  **Restart**

12: **end if**

13: Find *α* and *β* satisfy *αR*_*f*_ + *βR*_*g*_ = 1 by Extended Euclidean Algorithm, that is, (*αρ*_*f*_)*f* + (*βρ*_*g*_) *g* = 1 + *k*(*x*^*N*^ + 1).

14: Let *F* = *qβρ*_*g*_, *G* = −*qαρ*_*f*_, then *f* * *G* − *g* * *F* = *q* (mod *X*^*N*^ + 1)

15: Let $k=\lceil \frac{F\bar{f}+G\bar{g}}{f\bar{f}+g\bar{g}}\rfloor$, Reduce *F* and *G* as *F* ← *F* − *k* * *f*, *G* ← *G* − *k* * *g*.

16: **return** KGC’s public key *mpk* = *h* = *f*^−1^*g*, KGC’s secret key $msk=B=(\begin{array}{cc} A_{g} & -A_{f}\\ A_{G} & -A_{F} \end{array})$, where $A_{g},-A_{f},A_{G},-A_{F}$ are anti-circulant matrices whose *i*^*th*^ row consists of the coefficients of the polynomial *gx*^*i*^ mod (*X*^*N*^ + 1), *fx*^*i*^ mod (*X*^*N*^ + 1), *Gx*^*i*^ mod (*X*^*N*^+ 1) and *Fx*^*i*^ mod (*X*^*N*^ + 1), respectively.

### Address generation

The wallet address in blockchain technology is similar to the bank account number, which is one of the important components of blockchain. In this paper, to ensure the privacy of the recipient user, following the address generation model of Bitcoin, the address is not the public key but the hash of the public key. The address can be deduced from the public key, but the public key cannot be inferred back from the address because the hash function is a one-way function. A node address is generated as follows:

(1) Node runs Wallet Seed Key Generation **Algorithm 3** to output public key *h* = *f*^−1^*g* and a short basis
  $$\begin{array}{c} B=(\begin{array}{cc} A_{g} & -A_{f}\\ A_{G} & -A_{F} \end{array})\in Z_{q}^{2N\times 2N}\text{of}\underset{h,q}{⋀} \end{array}$$ (10)
  saved as the seed lattice basis in the wallet.
(2) The node randomly chooses subpublic keys **A**_**1**_, **A**_**2**_, …, **A**_**n**_ ∈ $Z_{q}^{N\times 1}$. Node concatenates matrices **A**_**1**_, **A**_**2**_, …, **A**_**n**_ behind *h*, denoted by $A_{1}^{{}^{\prime }}=h∥A_{1}$, $A_{2}^{{}^{\prime }}=h∥A_{2}$, …, and $A_{N}^{{}^{\prime }}=h∥A_{N}$, and sets them as the public keys for signature verification.
(3) The node runs algorithm *SampleGau*(**B**, *s*, **A**_**i**_) to generate private key tuples $S_{i}=\left(S_{i_{1}},S_{i_{2}}\right)$, where ($S_{i_{1}}$, $S_{i_{2}}$) satisfies $S_{i_{1}}+S_{i_{2}}h=A_{i}$ and $∥\left(S_{i_{1}},S_{i_{2}}\right)∥<s\sqrt{2N}$, which are used for transaction signing.
(4) Node maps matrices $A_{1}^{{}^{\prime }},A_{2}^{{}^{\prime }},\ldots ,A_{N}^{{}^{\prime }}$ into the corresponding vector **V**_**1**_, **V**_**1**_, …, **V**_**N**_.
(5) Node obtains *N* different addresses **Ad**_**1**_, **Ad**_**2**_, …, **Ad**_**N**_ through Secure Hash Algorithm (**SHA256**), RACE Integrity Primitives Evaluation Message Digest (**RIPEMD160**) algorithm and **Base58Check** encoding algorithm, that is, **Ad**_**i**_ = **Base58Check**(**RIPEMD160**(**SHA256**(**V**_**i**_))).

Simply providing the address does not allow others to learn the public key. As a rule, there is no security risk in making public keys public. In fact, if there are funds corresponding to an address, to spend the funds, the public key needs to be provided for signature verification. If an address has been traded at least once, the public key for that address is actually public.

### Transaction over lattice

The interaction process of nodes in the IoT is as follows:

(1) The node initiates the transaction *u* request.
(2) The node selects a pair of subpublic keys $A_{i}^{{}^{\prime }}$ and private keys $\left(S_{i_{1}},S_{i_{2}}\right)$ from its wallet.
(3) To prevent an attacker from forging a signature, the node signs the transaction *u* with private keys $\left(S_{i_{1}},S_{i_{2}}\right)$ from its wallet. The signature works as follows.

- Node selects a random **y**_**1**_, **y**_**2**_ $\in D_{\sigma }^{N}$.
- Node computes **c** = *H*_1_(**y**_**1**_ + **y**_**2**_*h*, *u*).
- Node computes $z_{1}=S_{i_{1}}c+y_{1}$, $z_{2}=S_{i_{2}}c+y_{2}$.
- A node generates the signature *Sig* = (**z**_**1**_,**z**_**2**_,**c**) with probability $min(\frac{D_{\sigma }^{N}}{MD_{\sigma ,S_{i}c}^{N}},1)$, where *M* = *O*(1). If nothing is output, repeat the above steps (approximately equal to 7).

(4) The transaction *u*, signature (**z**_**1**_,**z**_**2**_,**c**), Node’s A subpublic key $A_{i}^{{}^{\prime }}$ and Node’s B Address **Ad**_**Bi**_ are broadcast to all nodes.
(5) Each node verifies the transactions according to the following rules. Signature *Sig* = (**z**_**1**_,**z**_**2**_,**c**) on transaction *u* is valid if and only if $∥\left(z_{1},z_{2}\right)∥\leq 2\sigma \sqrt{N}$ and **c** = *H*_1_(*h* **z**_**2**_ + **z**_**1**_ − **A**_**i**_**c**, *u*).
(6) The verified transactions are stored in a block, which is locked (hashed) and becomes part of the blockchain permanently when the other nodes in the network have verified it.

## Correctness and security proof

### Correctness

**Theorem 1** *The proposed postquantum blockchain architecture for the IoT over the NTRU lattice satisfies correctness*.

**Proof 0.1** *According to the above construction, for any transaction u and public key* $A_{i}^{{}^{\prime }}$ , *we established the following formula*:

$$\begin{array}{l} H_{1}(hz_{2}+z_{1}-A_{i}c,u)\\ =H_{1}(h(S_{i_{2}}c+y_{2})+S_{i_{1}}c+y_{1}-(S_{i_{1}}+S_{i_{2}}h)c,u)\\ =H_{1}(y_{1}+y_{2}h,u)\\ =c \end{array}$$

*Therefore, we have* **c** = *H*_1_(*h* **z**_**2**_ + **z**_**1**_ − **A**_**i**_**c**, *u*) *satisfied. Then, by the result of* [37], *the resulting* **z**_**i**_ *distribution is* $D_{\sigma }^{N}$. As a result, by **Lemma 1**, *we have* $∥z_{i}∥\leq 2\sigma \sqrt{N}$ *with overwhelming probability, that is*, $∥\left(z_{1},z_{2}\right)∥\leq 2\sigma \sqrt{2N}$ *satisfied with overwhelming probability. Therefore, we can conclude that the signature scheme in our lattice-based blockcard scheme is correct*.

### Security

The security of our scheme is ensured by the following counterfactual:

**Theorem 2** Our postquantum blockchain architectures for IoT over the NTRU lattice are existential unforgeable against the adaptive chosen message and address attacks in the random oracle model.

**Proof 0.2** *We assume that there is a polynomial adversary* A , *and* A *can break our postquantum blockchain architectures for IoT over the NTRU lattice with nonnegligible probability. Based on the information obtained by adversary* mathcalA, *we can construct algorithm* mathcalC *to solve the SIS problem on the NTRU lattice with nonnegligible probability*.

Step 1 The algorithm C randomly selects secure hash function *H*_1_ = {0, 1}* → {**v**: **v** ∈ {−1, 0, 1}^*k*^, ∥**v** ∥ ≤λ} and matrix *h*, adversary A get the required parameters PP = {*H*_1_,**A**} from C.

Step 2 *Although we use double hash to ensure that the user’s public key is hidden under the address, when a node generates a transaction, the public key is necessarily published to the whole network and obtained by the adversary* A [40].

Step 3 *When* A *proposes H*_1_ *query on* (**y**_**1**_ + **y**_**2**_*h*, *u*). C *correspondingly looks H*_1_ − *list*, *which is* (**y**_**i1**_ + **y**_**i2**_*h*, *u*_*i*_, **c**_**i**_). If C *finds a matching pair of* (**y**_**1**_ + **y**_**2**_*h*, *u*, **c**), **c** *output in response. Otherwise*, C *randomly selects* **c** *from* {**v** ∈ {−1, 0, 1}^*k*^, ∥**v** ∥ ≤λ} and stores(**y**_**1**_ + **y**_**2**_*h*, *u*, **c**) *in* *H*_1_ − *list*, *and* **c** *is output in response*.

Step 4 *When* A *wants to get signify u on the* $A_{i}^{{}^{\prime }}$, A *propose query on* $\left(A_{i}^{{}^{\prime }},u\right)$. C *outputs Sig* = **(z**_**1**_**,z**_**2**_**,c)** *by running algorithm Sign*(*Par*, *u*, **S**_**i**_).

Step 5 *When adversary* A *is done with all desired queries, it outputs forgery* $\left(z_{1}^{{}^{\prime }},z_{2}^{{}^{\prime }},c^{{}^{\prime }}\right)$ *of address* **Ad**_**i**_ *on transaction u with nonnegligible probability. We can generate another valid signature* $\left(z_{1}^{*},z_{2}^{*},c^{*}\right)$ *according to the Forking lemma in* [41].

$$\begin{array}{c} H_{1}\left(hz_{2}^{{}^{\prime }}+z_{1}^{{}^{\prime }}-A_{i}c^{{}^{\prime }},u\right)=H_{1}\left(hz_{2}^{*}+z_{1}^{*}-A_{i}c^{*},u\right) \end{array}$$

*which implies that*

$$\begin{array}{c} hz_{2}^{{}^{\prime }}+z_{1}^{{}^{\prime }}-A_{i}c^{{}^{\prime }}=hz_{2}^{*}+z_{1}^{*}-A_{i}c^{*} \end{array}$$

*Since we have* $S_{i_{1}}+S_{i_{2}}h=A_{i}$ . *We obtain*

$$\begin{array}{c} \left(\begin{array}{c} h\\ 1 \end{array})(\begin{array}{cc} z_{2}^{{}^{\prime }}-z_{2}^{*}+S_{i_{2}}{}^{\prime }c^{{}^{\prime }}-S_{i_{2}}*c^{*} & z_{1}^{{}^{\prime }}-z_{1}^{*}+S_{i_{1}}{}^{\prime }c^{{}^{\prime }}-S_{i_{1}}*c^{*} \end{array}\right) \end{array}$$

$∥\left(z^{{}^{\prime }},z^{*}\right)∥\leq 2\sigma \sqrt{2N}$ *and* $∥\left(S_{i_{1}},S_{i_{1}}\right)∥\leq s\sqrt{2N}$ *with overwhelming probability. We obtain*

$$\begin{array}{c} ∥\left(z_{2}^{{}^{\prime }}-z_{2}^{*}+S_{i_{2}}{}^{\prime }c^{{}^{\prime }}-S_{i_{2}}*c^{*},z_{1}^{{}^{\prime }}-z_{1}^{*}+S_{i_{1}}{}^{\prime }c^{{}^{\prime }}-S_{i_{1}}*c^{*}\right)∥\leq \left(4\sigma +4s\lambda \right)\sqrt{2N} \end{array}$$

Step 5 *If* $(z_{2}^{{}^{\prime }}-z_{2}^{*}+S_{i_{2}}{}^{\prime }c^{{}^{\prime }}-S_{i_{2}}*c^{*},z_{1}^{{}^{\prime }}-z_{1}^{*}+S_{i_{1}}{}^{\prime }c^{{}^{\prime }}-S_{i_{1}}*c^{*})\neq 0$, *obviously, it is a solution to the SIS problem on the NTRU lattice. Now, we should prove that* $z^{{}^{\prime }}-z^{*}+S_{i}c^{*}-S_{i}c^{{}^{\prime }}\neq 0$ *with overwhelming probability. Since* $c^{*}\neq c^{{}^{\prime }}$. *Based on Property 4 of collision-resistant preimage sample functions* [37], *algorithm* C *can solve the SIS with a probability of at least* $(1-2^{\omega (\sqrt{\text{log}N})})\varepsilon$.

*According to the above analysis, if there is an adversary* A *breaking our scheme with nonnegligible probability, it can break the SIS problem over the NTRU lattice (NP-hard), which is obviously impossible*.

## Performance evaluation

Transaction size greatly affects the performance of the blockchain. In Bitcoin, for example, approximately 65% of the space is occupied by transaction data [42]. Therefore, we first compare our scheme with Yin et al.’s and Gao et al.’s pos quantum blockchain schemes [17, 19] in terms of blockchain transaction size. The results are shown in Table 1. *N* and λ are the security parameters of the blockchain system, m>5Nlogq, *σ* = 12λ*sN* and $M=\omega (\sqrt{\text{log}q})$, respectively. $\bar{s}=s\sqrt{(c+1)m}\omega \left(\sqrt{\text{log}n}\right)$.

**Table 1:** Comparison of the related postquantum blockchain.

	Lattice Type	Transaction Size
[17]	GPV Lattice	2Nmlog($\bar{s}\sqrt{3m}$)
[19]	GPV Lattice	N^2^logq
Ours	NTRU Lattice	2Nlog(12*σ*)+N(logλ+1)

To obtain a more intuitive comparison, we compare our solution with Yin et al.’s and Gao et al.’s solutions in terms of the concrete parameters, and the comparison results are shown in Table 2.

**Table 2:** Comparison in concrete parameters.

N	q	λ	Approximate Transaction Size(KB)		
			[17]	[19]	Ours
512	2^23^	14	143216	222	3
512	2^25^	14	162166	241	4
512	2^27^	28	187624	260	6

According to the results presented in Tables 1 and 2, the size of the transaction has been decreased with significative degree in the our blockchain scheme. Yin et al.’s transaction size is over 100000 KB, Gao et al.’s transaction size is over 200 KB, our transaction size is only a few KBs. The transaction size of our scheme smaller than Yin et al.’s and Gao et al.’s post quantum blockchain schemes.

Transaction size is closely related to the complexity of the blockchain system and blockchain performance, We can estimate blockchain performance by the following formula

$$\begin{array}{c} S=\left(V-v_{H}\right)/\left(T_{C}*v_{s}\right) \end{array}$$ (11)

where **S** denotes the throughput of the blockchain, **V** denotes the block capacity of the blockchain, **v**_**H**_ denotes the block header size, **T**_**C**_ denotes the time interval between two blocks, and **v**_**s**_ denotes the size of a single transaction.

Obviously, the complexity of our system is 2Nlog(12*σ*) + N(logλ + 1) which is smaller than Yin et al.’s and Gao et al.’s schemes. In the case of bitcoins, the block size is only 1024 KB, obviously, Yin et al.’s scheme easily exceeds the limits of resource-constrained environments, such as the IoT. As Yin et al. state in their article, their signatures are too large to make their scheme practical. Block in Gao et al.’s scheme only contains 4 transactions. Block in our scheme can contains 300 transactions. Our blockchain throughput is about 80 times faster than Gao et al.’s scheme.

Our scheme is expected to be used in resource-constrained environments, such as the IoT.

## Upgrade version

According to the Eq 11, to increase the throughput of the blockchain, the following methods can be used:

(1) The initial setting needs to set the block capacity **V** of the blockchain to the appropriate size and more so for public chains. To make changes to the block size later, it will inevitably create a hard fork problem, such as on August 1, 2017, when the Bitcoin network was hard forked into BCH and BTC, and at the same time, if the block capacity **V** is set is too large, it also inevitably leads to the loss of nodes with low storage capacity, resulting in the formation of centralization. Croman et al. [43] clearly indicates that in the current network environment, the upper limit of block capacity is 4M, and an excessively large value leads to the collapse of blockchain security.
(2) Reduce the time interval to generate blocks **T**_**C**_, which is essentially the design of a suitable consensus mechanism or underlying framework. The current consensus mechanisms are POW, POS, DPOS, PBFT, etc. POW can almost achieve complete decentralization, and security is also extremely high, which is also the most consistent with Matthew Wampler-Doty’s definition of decentralization. However, this type of consensus mechanism is bound to bring great resource consumption and time consumption, while POS and DPOS-like consensus mechanisms actually spread the responsibility of the center to nodes with high rights and interests, which provides a time reduction but also brings the question of centralization. Therefore, designing a consensus mechanism with adaptation according to Matthew Wampler-Doty’s definition of finding a balance between decentralization and practicality, is one of the research hotspots.
(3) Reducing the weight of signatures in the block, reducing the size of a single transaction **v**_**s**_ or reducing the size of the weight of signatures in the whole block can achieve speedups. In the process of upgrading GPV to an NTRU grid, we have reduced the size of a single transaction **v**_**s**_ substantially, and thus, the impractical quantum-resistant blockchain is optimized to be practical (near practical).

We briefly describe how the Segregated Witness and Aggregate Signature technologies can be used as soft fork upgrade solutions to change or reduce the weight of signatures and further optimize the blockchain network.

### Segregated witness

**Fig 3:**
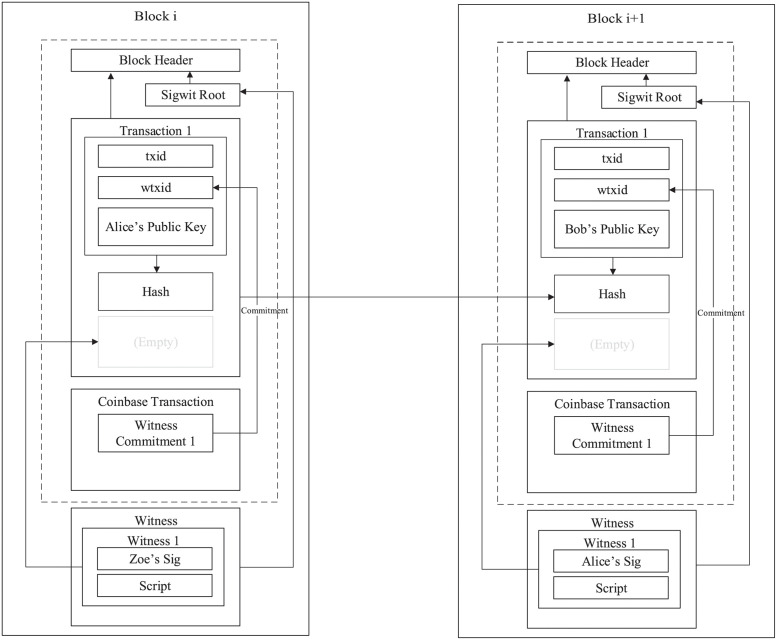
General framework of the Segregated Witness.

The Segregated Witness (SegWit) is a blockchain scaling technology in the engineering sense. The core of the Segregated Witness is to move the digital signature information of a transaction out of the block into a separate witness data structure that accompanies the transaction. This allows each block to carry more transactions (and there is truly no need to keep the digital signature inside the block once it has been verified), which indirectly improves blockchain performance. In the case of Bitcoin, for example, the signature data can occupy up to 65% of a block, and the isolated witness removes the signature data from the input of the transaction, increasing the effective block size from 1 MB to approximately 4 MB, allowing the Bitcoin blockchain to accept both new 4 MB blocks and 1 MB blocks through a clever engineering technique. In Fig 3, we present the general framework of the Segregated Witness.

### Aggregate signature

The Aggregate Signature is a blockchain scaling technology in the cryptographic sense that compresses multiple individual signatures into one compact signature. Given a set of user nodes ${\left\{N_{i}\right\}}_{i=1}^{i=k}$, message set *u*_*i*_, and *k* signatures for each message, the signature generator (which may be different or an untrusted third party, such as a miner) can aggregate those *k* signatures into a unique short one. During the verification phase, the miner only needs to verify the short signature, and its validation is equivalent to each of the original signatures. In practical use, we can choose many numbers of independent signatures for aggregation based on different rules or purposes. Of course, we can also aggregate all the signatures in the block into one short signature to minimize the capacity usage. We present our construction based on [44, 45]. In addition, the parameters are used in the previous scheme.

(1) Nodes ${\left\{N_{i}\right\}}_{i=1}^{i=k}$ generate their seed public keys ${\left\{h_{i}\right\}}_{i=1}^{i=k}$, short basis ${\left\{B_{i}\right\}}_{i=1}^{i=k}$, ${\left\{S_{N_{i1}},S_{N_{i2}}\right\}}_{i=1}^{i=k}$ and ${\left\{{Ad}_{N_{i}}\right\}}_{i=1}^{i=k}$, respectively.
(2) Nodes **N**_**i**_ initiate the transaction *u*_*i*_ request.
(3) Nodes **N**_**i**_ generate the signature **Sig**_**i**_ = **(z**_**i**_**, z**_**i+1**_**, c)** of the transaction *u*_*i*_.
(4) Aggregators aggregate multiple signatures ${\left\{{Sig}_{i}\right\}}_{i=1}^{i=k}$ into a single signature.

**Fig 4:**
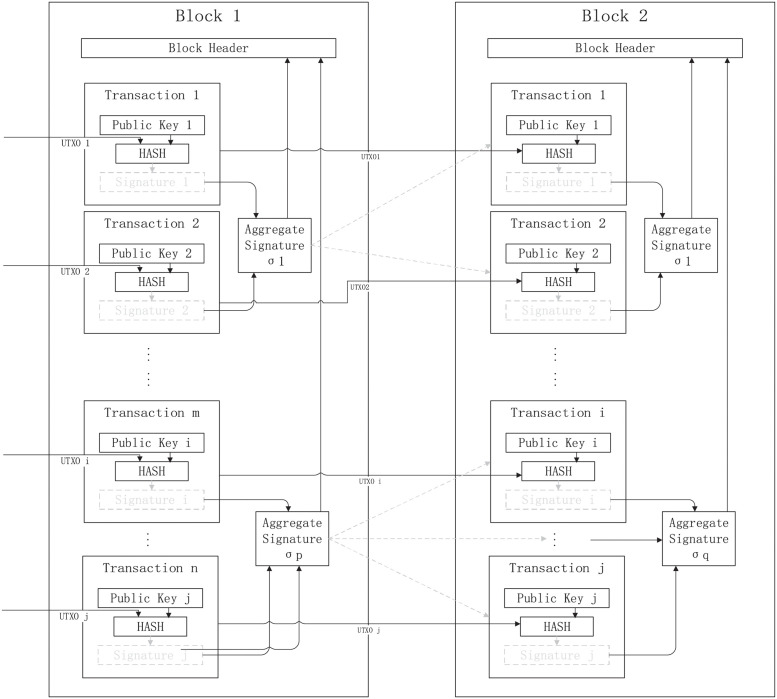
General framework of the Aggregate Signature in blockchain.

In Fig 4, we present the general framework of the Aggregate Signature.

## Conclusion

IoT technology faces many challenges, and blockchain technology may offer new possibilities for solving these problems. However, the current proposed blockchain-based IoT architectures are based on the traditional number theory problem, which is insecure against quantum analysis. Some quantum-resistant blockchain schemes cannot be applied in resource-constrained environments due to excessive signature size. In this paper, we proposed a NTRU lattice-based blockchain system for IoT which can be deployed over existing classical channels. We presented a new seed key generation algorithm to generate the sub-private keys for verifying the transaction message. The security proof shows that the our scheme is secure against the quantum computing attacks. The experimental results show that our NTRU-based blockchain system is more efficient than the existing scheme and expected to be applied in resource-constrained environments. Furthermore, to further improve the performance of the blockchain, we provide the basic framework of two improvement schemes, the Segregated Witness and the Aggregate Signature, over the NTRU lattice.

There are some open problems which are attractive to be explored further. First of all, blockchain is a data storage technology that can only be attached and cannot be deleted, with the continuous growth of blockchain, IoT devices can hardly have enough storage space, how to compress block content without affecting security will be an interesting research direction. Secondly, ECDSA and other signatures widely used in the blockchain are only about 40 bytes in size, but the best lattice signature scheme is still a few kilobytes, it is clear that in order for it to replace widely used number-theoretic primitives, lattice-based signature must be designed to be similarly efficient as ECDSA. Thirdly, Blockchain is an open database. This feature is crucial to the realization of non-repudiation mechanism, but it will bring privacy protection problems to the Internet of Things. Attackers can obtain the hidden associations by analyzing the information of blockchain nodes; therefore, it is necessary to research how to introduce privacy protection technologies into the above system and adopt zero knowledge proof and homomorphic encryption technology to improve the privacy protection capability of blockchain-based system.

## References

[1] Rahman MA, Rashid MM, Hossain MS, Hassanain E, Alhamid MF, Guizani M. Blockchain and IoT-based cognitive edge framework for sharing economy services in a smart city. IEEE Access. 2019;7:18611–18621. doi: 10.1109/ACCESS.2019.2896065

[2] Fayoumi AG, Hajjar AF. Advanced learning analytics in academic education: Academic performance forecasting based on an artificial neural network. International Journal on Semantic Web and Information Systems (IJSWIS), 2020, 16(3): 70–87. doi: 10.4018/IJSWIS.2020070105

[3] Atlam HF, Alenezi A, Alharthi A, Walters RJ, Wills GB. Integration of cloud computing with internet of things: challenges and open issues. In: 2017 IEEE International Conference on Internet of Things (iThings) and IEEE Green Computing and Communications (GreenCom) and IEEE Cyber, Physical and Social Computing (CPSCom) and IEEE Smart Data (SmartData). IEEE; 2017. p. 670–675.

[4] Nguyen DC, Pathirana PN, Ding M, Seneviratne A. Integration of Blockchain and Cloud of Things: Architecture, Applications and Challenges. arXiv preprint arXiv:190809058. 2019;.

[5] Sharma R, Sharma N. Attacks on Resource-Constrained IoT Devices and Security Solutions. Applications and Challenges. International Journal of Software Science and Computational Intelligence (IJSSCI), 2022, 14(1): 1–21. doi: 10.4018/IJSSCI.310943

[6] Dive R. Global Blockchain IoT Market Insight and Outlook 2026; 2020. https://www.researchdive.com/45/blockchain-iot-market.

[7] Arsyad AA, Widayat IW, Koppen M. Supporting farming smart documentation system by modular blockchain solutions. Decision Making: Applications in Management and Engineering, 2022, 5(1): 1–26.

[8] Raj A, Prakash S. A Privacy-Preserving Authentic Healthcare Monitoring System Using Blockchain International Journal of Software Science and Computational Intelligence (IJSSCI), 2022, 14(1): 1–23. doi: 10.4018/IJSSCI.310942

[9] Gupta BB, Li KC, Leung VCM. Blockchain-assisted secure fine-grained searchable encryption for a cloud-based healthcare cyber-physical system. IEEE/CAA Journal of Automatica Sinica, 2021, 8(12): 1877–1890. doi: 10.1109/JAS.2021.1004003

[10] Lu J, Shen J, Vijayakumar P. Blockchain-based secure data storage protocol for sensors in the industrial Internet of Things IEEE Transactions on Industrial Informatics, 2021, 18(8): 5422–5431. doi: 10.1109/TII.2021.3112601

[11] Ding S, Cao J, Li C, Fan K, Li H. A novel attribute-based access control scheme using blockchain for IoT. IEEE Access. 2019;7:38431–38441. doi: 10.1109/ACCESS.2019.2905846

[12] Sharma PK, Chen MY, Park JH. A software defined fog node based distributed blockchain cloud architecture for IoT. Ieee Access. 2017;6:115–124. doi: 10.1109/ACCESS.2017.2757955

[13] Fedorov AK, Kiktenko EO, Lvovsky AI. Quantum computers put blockchain security at risk; 2018. 10.1038/d41586-018-07449-z 30451981

[14] Kiktenko EO, Pozhar NO, Anufriev MN, Trushechkin AS, Yunusov RR, Kurochkin YV, et al. Quantum-secured blockchain. Quantum Science and Technology. 2018;3(3):035004. doi: 10.1088/2058-9565/aabc6b

[15] Iovane G. MuReQua Chain: Multiscale Relativistic Quantum Blockchain. IEEE Access. 2021;9:39827–39838. doi: 10.1109/ACCESS.2021.3064297

[16] Li CY, Chen XB, Chen YL, Hou YY, Li J. A New Lattice-Based Signature Scheme in Post-Quantum Blockchain Network. IEEE Access. 2018;7:2026–2033. doi: 10.1109/ACCESS.2018.2886554

[17] Yin W, Wen Q, Li W, Zhang H, Jin Z. An anti-quantum transaction authentication approach in blockchain. IEEE Access. 2018;6:5393–5401. doi: 10.1109/ACCESS.2017.2788411

[18] Ma C, Jiang M. Practical lattice-based multisignature schemes for blockchains. IEEE Access. 2019;7:179765–179778. doi: 10.1109/ACCESS.2019.2958816

[19] Gao YL, Chen XB, Chen YL, Sun Y, Niu XX, Yang YX. A secure cryptocurrency scheme based on post-quantum blockchain. IEEE Access. 2018;6:27205–27213. doi: 10.1109/ACCESS.2018.2827203

[20] Raavi M, Wuthier S, Chandramouli P, Balytskyi Y, Zhou X, Chang SY. Security comparisons and performance analyses of post-quantum signature algorithms. In: International Conference on Applied Cryptography and Network Security. Springer; 2021. p. 424–447.

[21] Da Xu L, Lu Y, Li L. Embedding blockchain technology into IoT for security: A survey. IEEE Internet of Things Journal. 2021;8(13):10452–10473. doi: 10.1109/JIOT.2021.3060508

[22] Sun Y, Zhang L, Feng G, Yang B, Cao B, Imran MA. Blockchain-enabled wireless Internet of Things: Performance analysis and optimal communication node deployment. IEEE Internet of Things Journal. 2019;6(3):5791–5802. doi: 10.1109/JIOT.2019.2905743

[23] Grover LK. Quantum mechanics helps in searching for a needle in a haystack. Physical review letters. 1997;79(2):325. doi: 10.1103/PhysRevLett.79.325

[24] Xie J, Hu Yp, Gao Jt, Gao W. Efficient identity-based signature over NTRU lattice. Frontiers of Information Technology & Electronic Engineering. 2016;17(2):135–142. doi: 10.1631/FITEE.1500197

[25] Kolias C, Kambourakis G, Stavrou A, Voas J. DDoS in the IoT: Mirai and other botnets. Computer. 2017;50(7):80–84. doi: 10.1109/MC.2017.201

[26] Javaid U, Siang AK, Aman MN, Sikdar B. Mitigating loT device based DDoS attacks using blockchain. In: Proceedings of the 1st Workshop on Cryptocurrencies and Blockchains for Distributed Systems; 2018. p. 71–76.

[27] Chen M, Tang X, Cheng J, Xiong N, Li J, Fan D. A DDoS attack defense method based on blockchain for IoTs devices. In: International Conference on Artificial Intelligence and Security. Springer; 2020. p. 685–694.

[28] Ouaddah A, Elkalam AA, Ouahman AA. Towards a novel privacy-preserving access control model based on blockchain technology in IoT. In: Europe and MENA cooperation advances in information and communication technologies. Springer; 2017. p. 523–533.

[29] Yang Q, Wang H. Privacy-preserving transactive energy management for IoT-aided smart homes via blockchain. IEEE Internet of Things Journal. 2021;8(14):11463–11475. doi: 10.1109/JIOT.2021.3051323

[30] Kumar P, Kumar R, Srivastava G, Gupta GP, Tripathi R, Gadekallu TR, et al. PPSF: a privacy-preserving and secure framework using blockchain-based machine-learning for IoT-driven smart cities. IEEE Transactions on Network Science and Engineering. 2021;8(3):2326–2341. doi: 10.1109/TNSE.2021.3089435

[31] Liu S, Yu J, Xiao Y, Wan Z, Wang S, Yan B. BC-SABE: Blockchain-aided searchable attribute-based encryption for cloud-IoT. IEEE Internet of Things Journal. 2020;7(9):7851–7867. doi: 10.1109/JIOT.2020.2993231

[32] Pan J, Wang J, Hester A, AlQerm I, Liu Y, Zhao Y. EdgeChain: An edge-IoT framework and prototype based on blockchain and smart contracts. IEEE Internet of Things Journal. 2018;6(3):4719–4732. doi: 10.1109/JIOT.2018.2878154

[33] Huh S, Cho S, Kim S. Managing IoT devices using blockchain platform. In: 2017 19th international conference on advanced communication technology (ICACT). IEEE; 2017. p. 464–467.

[34] Liu B, Yu XL, Chen S, Xu X, Zhu L. Blockchain based data integrity service framework for IoT data. In: 2017 IEEE International Conference on Web Services (ICWS). IEEE; 2017. p. 468–475.

[35] Zhao Q, Chen S, Liu Z, Baker T, Zhang Y. Blockchain-based privacy-preserving remote data integrity checking scheme for IoT information systems. Information Processing & Management. 2020;57(6):102355. doi: 10.1016/j.ipm.2020.102355

[36] Rejeb A, Keogh JG, Treiblmaier H. Leveraging the internet of things and blockchain technology in supply chain management. Future Internet. 2019;11(7):161. doi: 10.3390/fi11070161

[37] Lyubashevsky V. Lattice signatures without trapdoors. In: Annual International Conference on the Theory and Applications of Cryptographic Techniques. Springer;. p. 738–755.

[38] Cai J, Nerurkar A. Approximating the svp to within a factor 1/dim” is NP hard under randomized reductions. In: Proceedings of the 38th IEEE Conference on Computational Complexity;. p. 46–55.

[39] Awange JL, Paláncz B. Polynomial resultants. In: Geospatial Algebraic Computations. Springer; 2016. p. 53–68.

[40] Narayanan A, Bonneau J, Felten E, Miller A, Goldfeder S. Bitcoin and cryptocurrency technologies: a comprehensive introduction. Princeton University Press; 2016.

[41] Pointcheval D, Stern J. Security arguments for digital signatures and blind signatures. Journal of cryptology. 2000;13(3):361–396. doi: 10.1007/s001450010003

[42] App C. The Difference Between BTC and BCH: Explained; 2018. https://medium.com/coinstack-app/the-difference-between-btc-and-bch-explained-3e51bdc0f05e.

[43] Croman K, Decker C, Eyal I, Gencer AE, Juels A, Kosba A, et al. On scaling decentralized blockchains. In: International conference on financial cryptography and data security. Springer; 2016. p. 106–125.

[44] El Bansarkhani R, Buchmann J. Towards lattice based aggregate signatures. In: International Conference on Cryptology in Africa. Springer; 2014. p. 336–355.

[45] Jia X, Yupu H, Juntao G, JIANG M, et al. Certificateless Sequential Aggregate Signature Scheme on NTRU Lattice. Chinese Journal of Electronics. 2019;28(2):294–300. doi: 10.1049/cje.2019.01.019

